# Overexpression of DUSP6 enhances chemotherapy-resistance of ovarian epithelial cancer by regulating the ERK signaling pathway

**DOI:** 10.7150/jca.37267

**Published:** 2020-03-04

**Authors:** Yan Gao, Hui Li, Qing Han, Yuan Li, Tongxia Wang, Cuiyu Huang, Yiqing Mao, Xi Wang, Qun Zhang, Junrui Tian, David M. Irwin, Huanran Tan, Hongyan Guo

**Affiliations:** 1 Department of Obstetrics and Gynecology, Peking University Third Hospital, Beijing,; 2 Department of Pharmacology, School of Basic Medical Sciences, Peking University Health Science Center, Beijing,; 3 Department of Laboratory Medicine and Pathobiology, University of Toronto, Toronto, Canada.

**Keywords:** DUSP6, ERK signaling pathway, side population cell, ovarian epithelial cancer, chemotherapy resistance

## Abstract

**Objective**: DUSP6 is a negative regulator of the ERK signaling pathway and plays an important role in chemotherapy-resistance. Previously we showed that DUSP6 is overexpressed in ovarian cancer side population (SP) cells that possess cancer stem cell-like properties and are quiescent and chemotherapy-resistant. Here, we explore the effects of DUSP6 on chemotherapy-resistance by examining its regulation of the ERK signaling pathway and G0/G1 cell cycle arrest.

**Methods**: mRNA and protein expression of DUSP6 and G0/G1 cell cycle checkpoint regulating proteins (CyclinD1, CyclinD3 and CyclinE2) was evaluated among ovarian cancer cell lines and tissue samples. Ovarian cancer cells were transiently transfected to overexpress DUSP6. After treatment with cisplatin, cell viability was measured by the MTS assay at 48 hours and the half maximal inhibitory concentration (IC50) for each cell line was calculated. Subcellular localization and cell cycle analysis were determined by using immunofluorescence and FACS, respectively.

**Results**: SKOV3 and OVCAR8 SP cells were shown to express higher levels of DUSP6 and lower levels of CyclinD3 compared with non-SP (NSP) cells (P<0.001). Among 39 ovarian cancer tissue samples, expression of DUSP6 in the chemotherapy-resistant group (12 samples) was higher than in the chemotherapy-sensitive group (27 samples) (P<0.05). While a lower level of expression of CyclinD3 was seen in the chemotherapy-resistant group, it was not statistically different from the chemotherapy-sensitive group. HO8910 cells where shown to have higher IC50 to cisplatin than SKOV3 or OVCAR8 cells, and this correlated with higher levels of DUSP6 expression. Overexpression of DUSP6 in SKOV3 cells led to an increase in cisplatin IC50 values (P<0.05), and also markedly reduced the expression levels of phospho-ERK1/2 and CyclinD3 and to the predominance of cells in the G0/G1 phase.

**Conclusion**: Our findings reveal an enhancement of chemotherapy-resistance and a predominance of cells in G1 cell cycle arrest in DUSP6-overexpressing ovarian cancer cells. This suggests that overexpression of DUSP6 promotes chemotherapy-resistance through the negative regulation of the ERK signaling pathway, increasing the G0/G1 phase ratio among ovarian cancer cells, and leading to cellular quiescence.

## Introduction

Epithelial ovarian cancer (EOC) is the most lethal gynecologic malignancy and commonly displays tumor recurrence and chemotherapy-resistance^1^. Surgery followed by chemotherapy is the primary initial treatment in most advanced-stage patients, where the current treatment with cisplatin, in combination with paclitaxel, results in complete remission in 80% of patients^2^-^3^. Unfortunately, remission is usually short lived with subsequent recurrence due to chemotherapy-resistance, and death as a consequence of metastatic spread^3^. Presently, emerging evidence suggests that a small group of tumor cells, termed cancer stem cells (CSC), survive the debulking surgery and by remaining quiescent through the following chemotherapy become available to trigger tumorigenesis and chemotherapy- resistance^4^-^8^. Using flow cytometry and Hoechst 33342 efflux staining a small portion of the ovarian cancer cells can be isolated, which are known as side population (SP) cells^9^-^11^. These cells have been shown to harbor cancer stem cell-like properties and potentially contribute to chemotherapy-resistance^9^-^15^.

RNA‑sequencing (RNA‑seq) is a recently developed method for transcriptome profiling that employs next‑generation sequencing technologies^16^. This approach has been extensively employed to investigate mechanisms of drug resistance in various types of cancers, which has led to the identification of differentially expressed genes that provide insight into novel complex mechanisms of resistance to anticancer drugs^16^-^18^. Here we used RNA-seq to identify genes that are differentially expressed between human ovarian SKOV3 SP and NSP cells, genes that might underlie chemotherapy-resistance in ovarian cancer.

DUSP6 is a member of a subfamily of protein tyrosine phosphatases known as dual-specificity phosphatases (DUSPs), which dephosphorylates extracellular signal-regulated protein kinase 1/2 (ERK1/2) to negatively regulate ERK signaling^19^,^20^. Through its regulation of ERK signaling it modulates cell proliferation, differentiation and apoptosis^21^-^24^. DUSP6 has been reported to be overexpressed in the ocular surface side population stem cells that possess a quiescent and slow cycling phenotype^25^-^27^. Many studies have confirmed a role for DUSP6 in the negative regulation of ERK signaling pathway and the reduction in cellular proliferation rates^19^,^20^. Studies have shown that higher levels of DUSP6 expression are seen in relatively inactive tumor cells compared with actively proliferating tumor cells^28^,^29^. Antitumor drugs such as cisplatin mainly kill highly proliferating tumor cells, while quiescent tumor cells are usually resistant^7^. These observations raise the hypothesis that DUSP6 plays an important role in chemotherapy- resistance by causing cellular quiescence through its regulation of the ERK signaling pathway.

In this study we analyzed the expression of DUSP6 in SP and NSP cells, where it is differentially expressed, and from chemotherapy-resistant or -sensitive ovarian cancer cell lines to deduce the role of DUSP6 in negatively regulating ERK1/2 activity during the cell cycle, which leads to G0/G1 arrest and chemotherapy-resistance.

## Materials and Method

### Clinical samples and cell lines

Patients with stages IIIC or IV defined by laparoscopy or laparotomy by the International Federation of Gynecology and Obstetrics (FIGO) guidelines and who underwent additional six cycles of platinum-based chemotherapy were included in this study. However, patients refused to complete chemotherapy after surgery or were found to have complications with other cancers were excluded. Surgically resected primary ovarian cancer tissue samples were collected from thirty-nine patients for this study (see Supplementary Table 1). Ethical approval for the collection of clinical specimens was obtained from the Peking University Health Science Center ethics committee. In addition, four ovarian cancer cell lines, HO8910, OVCAR8, SKOV3, and OVCAR3 were used in this study, and were obtained from the Cell Bank of Type Culture Collection of the Chinese Academy of Sciences (Shanghai, China). HO8910, OVCAR8 and SKOV3 cells were cultured in high glucose Dulbecco's modified Eagle's Medium (DMEM, GIBCO) supplemented with 10% FBS (HyClone), 10mM HEPES (Amresco), 100 U/ml penicillin (Sigma) and 100 μg/ml streptomycin (Sigma) and maintained at 37°C and 5% CO_2_. OVCAR3 cells were cultured in RPMI-1640 supplemented with 2.0 g glucose and 0.3 g L-glutamine, as well as with 10% fetal bovine serum (FBS), 1% penicillin/ streptomycin. Cells were used within 7 days for sorting for SP cells by flow cytometry, transient transfection, RT-PCR or immunoassays.

### Identification of SP cells

SP cells were isolated from SKOV3 and OVCAR8 cells as previously described^11^-^14^. Briefly, cultured SKOV3 and OVCAR8 cells were stained with 5 mg/ml Hoechst 33342 dye (Sigma) by incubating for 90 min with and without 50 mM verapamil. Cell samples were analyzed and sorted using a FASCalibur flow cytometry (Becton Dickinson, Mountain View, CA, USA). SP cells have low Hoechst staining, while NSP cells have high levels of staining. Flow cytometry was performed by the Peking University Health Science Center Central Laboratory.

### Transcriptome sequencing and analysis

Transcriptome analysis was used to identify differentially expressed genes in SP and NSP cell from the SKOV3 cells respectively. For cDNA library construction, approximately 5μg of the total RNA per sample was used for RNA sample preparations. A total of 3 libraries were constructed. Libraries for sequencing were generated using an IlluminaTruSeq RNA Sample Preparation Kit (Illumina, San Diego, CA, USA). Transcriptome sequencing, conducted by Novogene (Beijing, China), used the paired end approach and was performed on the IlluminaHiSeq 2500 platform using manufacturer's protocol, and generated approximately 125 bp paired end (PE) raw reads. Clean reads from the three transcriptome libraries were obtained from the raw data by filtering adaptor and low-quality read sequences. The remaining clean reads were assembled using Trinity software for de novo transcriptome assembly without a reference genome. The quality of the assembly was assessed by Novogene before subsequent analyses. All non-redundant sequences were annotated against the protein family (Pfam), KEGG Ortholog database (KEGG) and GeneOntology (GO) databases. The expression level of each transcript was measured as the number of clean reads mapped to its sequence and expressed as RPKM (Reads Per Kilobase of transcript per Million mapped reads) using RSEM 1.2.3. FDR threshold was determined using DESeq. FDR<0.05 and fold change >2 were used to identify differentially expressed genes.

### RNA extraction and RT-PCR

Total RNA, from the ovarian cancer cell lines described above, tumor tissues, SKOV3 SP and NSP cells, OVCAR8 SP and NSP cells, was extracted using TRIZOL. cDNA was synthesized using PrimerScript® 1st Strand cDNA Synthesis Kits (Takara, cat # D6110A). Quantitative detection of mRNA levels for actin, DUSP6, CyclinD1, CyclinD3, and CyclinE2 genes was performed with the StepOne™ System with PowerUpTM SYBR® GreenMasterMix (applied biosystems) according to the manufacturer's instructions. Primers for each gene for RT-PCR (see Supplementary Table 2) were synthesized by TSINGKE. PCR amplification was carried out in a total volume of 20 μL, containing 1 μL cDNA solution, 10 μL of 2ⅹPowerUpTM SYBR® GreenMasterMix, 1 μL each primer at 5 μM, 7 μL of nuclease-free water. GAPDH was quantified and used for the normalization of expression values of the other genes. Fluorescence signals measured during the amplification were considered positive if the fluorescence intensity was more than 20-fold greater than the standard deviation of the baseline fluorescence. The ΔΔCT method of relative quantification was used to determine the fold change in expression. Here, threshold cycle (CT) values of the target mRNAs were first normalized to the CT values of the internal control, β-actin, in the same samples (ΔCT = CTtarget - CTcon), and then further normalized with the internal control (16-week-old gck^w/w^ mice were used as internal control) (ΔΔCT = ΔCT - ΔCTcon). Fold change in expression was then obtained (2-ΔΔCT) as described^25^, ^26^. PCR conditions were as follows: 50°C for 120 s, 95°C for 120 s, 40 cycles at 95°C for 30s, 58°C for 30 s and extension at 72°C for 60 s using StepOne™ Real-Time PCR System. Comparative Ct method (2-(△△Ct) method) was used to analyze relative gene expressions with GAPDH as the internal control.

### Plasmids and Transfection

YFP-tagged DUSP6-expression construct, pcDNA3.1-DUSP6-YFP and the YFP-tagged empty vector construct, pcDNA3.1-YFP were provided by Professor Huanran Tan's lab, Peking University Health Science Center. To confirm expression of the introduced coding sequences, SKOV3 cells were transfected with the expression plasmid and YFP expression was visualized by confocal immunofluoresence. Cells were viewed in 5 different fields under the microscope, with the total number of cells and the numbers of cells with yellow fluorescence counted. The transfection rate was calculated by the following formula.


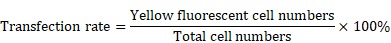


Briefly, 2 × 10^5^ SKOV3 cells were placed into each well of a 6-well plate (Corning) and cultured overnight. Cells were transfected with the plasmid using Neofect™ (Neofect Biotechnologies, Beijing, China) and cultured for 36h before visualization. Expression was confirmed using extracts from these cells by quantitative real-time polymerase chain reaction (RT-PCR) using DUSP6-specific primers and western blotting using a specific antibody (DUSP6, ab54940, Abcam).

### Immunofluorescence

Prior to staining for immunofluorescence, cells were allowed to adhere to the glass bottom of confocal dishes for 3 hours. Cells were then washed 3 times (5 min each) with phosphate buffered saline (PBS, pH 7.2), fixed in 4% paraformaldehyde in PBS containing 0.1% (vol/vol) Triton X-100 (PBST) at room temperature for 15 min and then washed again in PBS 3 times (5 min each). Cells were blocked with 5% bovine serum albumin (BSA, Boehringer) in PBST for 30 min at 37°C. Primary antibodies (DUSP6, ab54940, Abcam, pERK, 4370S, CST, tERK (ERK1+ERK2), ab184699, Abcam) were diluted to 1:40 in blocking solution. 200uL of the primary antibody solutions were added to each dish, and cells were incubated overnight at 4°C. After incubation, cells were washed 3 times (5 min each) with PBS. All subsequent steps were performed in the dark. 200μl of secondary antibodies (TRITC goat anti-mouse IgG, ZF-0313, FITC goat anti-rabbit IgG, ZF-0311, TRITC goat anti-rabbit IgG, ZF-0314,1:50 dilution in blocking solution) was added to each dish and incubated for 60 min at room temperature. After incubation, excess secondary antibodies were removed and 200μl of DAPI (C0065, Solarbio) was added to each dish and incubated for 10 min at room temperature in the dark. Cells were then washed 3 times (5 min each) with PBS and observed under a confocal microscope (Leica).

### Western blot analysis

Cell lysates were obtained from cells that were extensively washed with PBS and lysed directly in cell lysate solution. Protein concentration was determined using the BCA Protein Assay Kit. Equal amounts of protein (100 μg/lane) from cell lysates or culture medium were separated by sodium dodecyl sulfate polyacrylamide gel electrophoresis (SDS-PAGE) or Tricine-SDS-PAGE and transferred to polyvinylidene difluoride membranes (Hybond-P; GE Healthcare). Blots were probed with an appropriate primary antibody, followed by HRP-conjugated anti-rabbit IgG (Cell Signaling Technology, CST). Protein bands were visualized using an enhanced chemiluminescence (ECL) detection method (Bio-Rad), and band intensity was analyzed with a densitometer (LAS- 4000; GE Healthcare). Immunoreactive protein content of each sample was calculated based on a standard curve constructed using BSA. Each set of experiments was repeated at least 3 times to confirm the results. The level of GAPDH protein, measured by quantitative Western blotting using GAPDH antibody (ab181602), was used as an extraction and loading control.

### Cell viability

Cell viability was measured by the MTS colorimetric assay. Experiments were performed in triplicate for each time point. Cisplatin (CDDP) was diluted into culture medium to yield different concentrations and added to ovarian cancer cell lines for 48 hours at 37°C. New media, 100μl per well, was given to the cells with 20μl CellTiter 96® AQueous One Solution (MTS; Promega) added and incubated for 2 to 4 hours at 37°C. Absorbance was taken at 490 nm on a microplate spectrophotometer, with higher absorbance values correlating with greater viability. Background readings (reagents and media alone in empty wells) were subtracted from the absorbance.

### Cell Cycle Analysis

SKOV3 were harvested 36 h after transient transfection and fixed with 70% ethanol for 24 h. Cells were washed with PBS, stained with 20 μg/ml propidium iodide and 1 mg/ml RNase (Type IIA; Sigma), and collected on a Life Sciences Research flow cytometer configured with cellquest pro software (BD Biosciences, Franklin Lakes, NJ).

## Results

### SKOV3 SP and NSP cell transcriptomes and identification of differentially expressed genes

**Figure 1:**
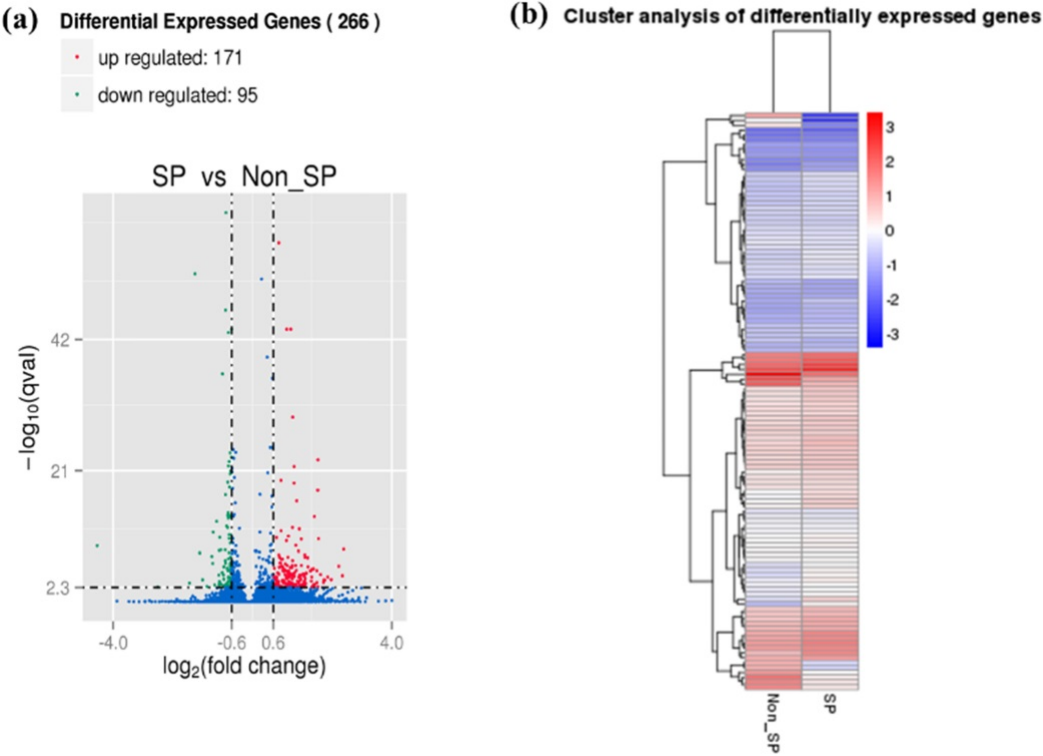
Differential expression of genes between SKOV3 SP and NSP cells. (a) Volcano Plot of differentially expressed genes. Blue refers to all genes, with red dots referring to genes with upregulated expression in SP cells, compared with NSP cells, and green dots refer to genes with downregulated expression in SP cells. (b) Heat map of the differential expression of the top 266 differentially expressed genes (171 upregulated (red) and 95 downregulated (blue)).

SKOV3 SP and NSP cell populations were isolated as previously described^13^. mRNA transcriptomes were generated for both cell populations. A total of 14,034 genes were detected, with 13,646 genes in the SP cells and 13,396 genes in the NSP cells. To identify differences in the gene expression profiles between the SP and NSP cells, we used edgeR to detect differentially expressed genes between the SKOV3 SP and NSP cells using previously described criteria^30^. A total of 13,008 genes exhibited differential expression between the SKOV3 SP and NSP cells, and when a fold change of >2 and a false positive rate of P<0.05 were applied, a total of 266 genes were identified as being differentially expressed. This total included 171 genes that were up-regulated in the SKOV3 SP cells and 95 genes that were down-regulated (Figure 1).

### Analysis of differentially expressed genes

**Figure 2:**
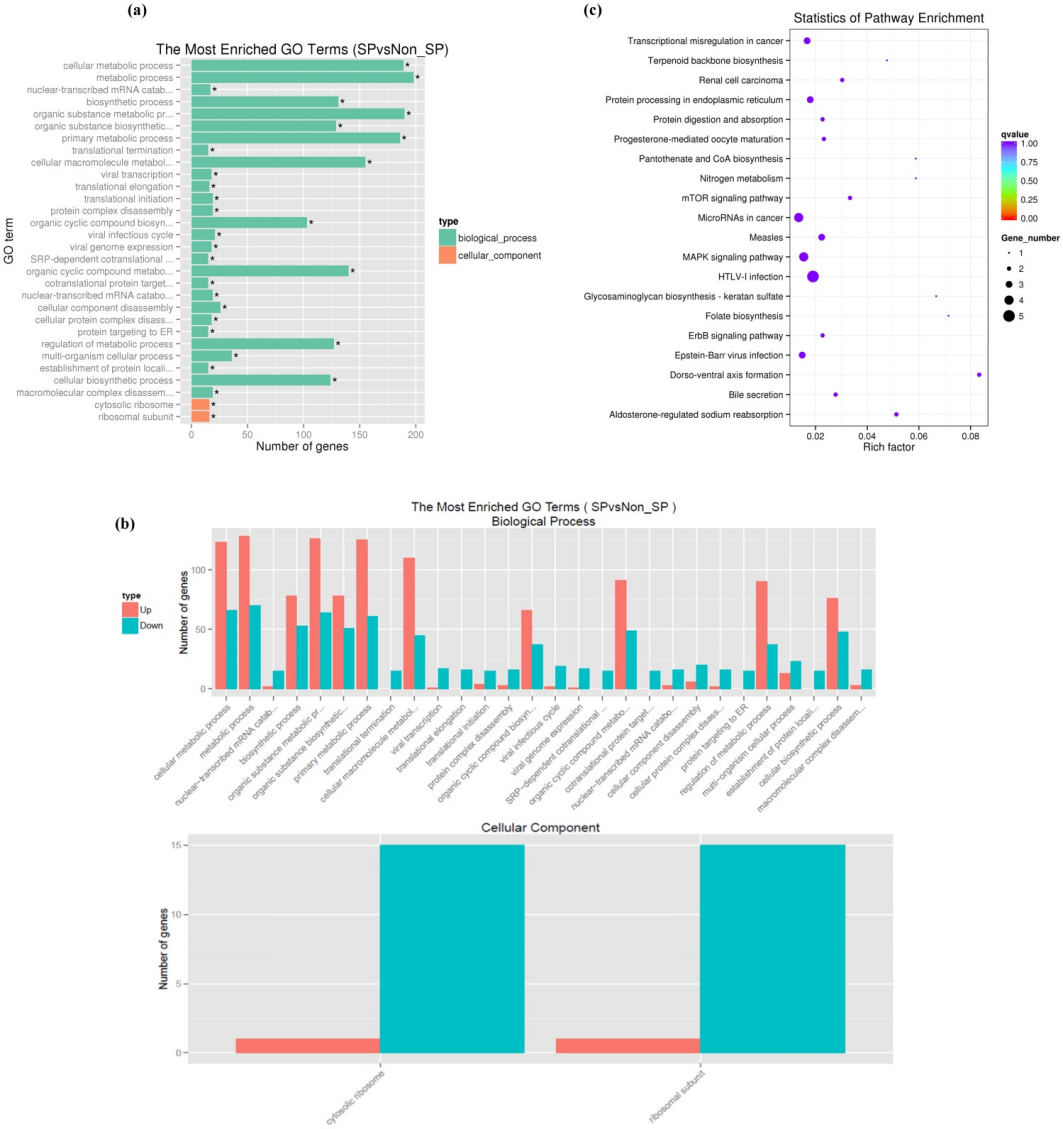
Functional enrichment of differentially expressed genes in SP cells. (a, b) GO analysis of the biological processes and cellular component terms. (c) KEGG analysis of the enrichment of signaling pathways possessing differentially expressed genes between SKOV3 SP and NSP cells.

To functionally analyze the differentially expressed genes, we used the Database for Annotation, Visualization and Integrated Discovery (DAVID) database^31^. Using a GO analysis, the biological process and cellular component terms were identified. The GO analysis of the biological processes demonstrated that the differentially expressed genes were enriched in biological process terms 'cellular metabolic process (198 genes), 'organic substance metabolic process' (190 genes) and 'cellular biosynthetic process' (124 genes). In addition, differentially expressed genes were also enriched in 'cellular macromolecule metabolic process' (156 genes), 'primary metabolic process' (186 genes), 'gene expression' (107 genes) and 'organic cyclic compound metabolic process' (80 genes) (Fig. 2 a, b). GO analysis of the cellular components demonstrated that the differentially expressed genes were primarily associated with cellular component terms 'intracellular organelle' (142 genes), 'cytoplasmic part' (124 genes), 'cytosolic part' (19 genes), 'ribosomal subunit' (16 genes) and cytosolic ribosome (16 genes) (Fig. 2 a, b). The enrichment of signaling pathways possessing differentially expressed genes was examined by a KEGG pathway analysis. Our results demonstrate that pathways with differentially expressed genes included 'metabolic pathways' (19 genes), 'HTLV-I infection' (13 genes), 'microRNAs in cancer' (10 genes), 'MAPK signaling pathway' (7 genes), 'transcriptional misregulation in cancer' (6 genes), 'PI3K-Akt signaling pathway' (9 genes) and 'cell cycle' (5 genes) (Fig. 2c).

### DUSP6 is overexpressed in ovarian cancer SP cells

**Figure 3:**
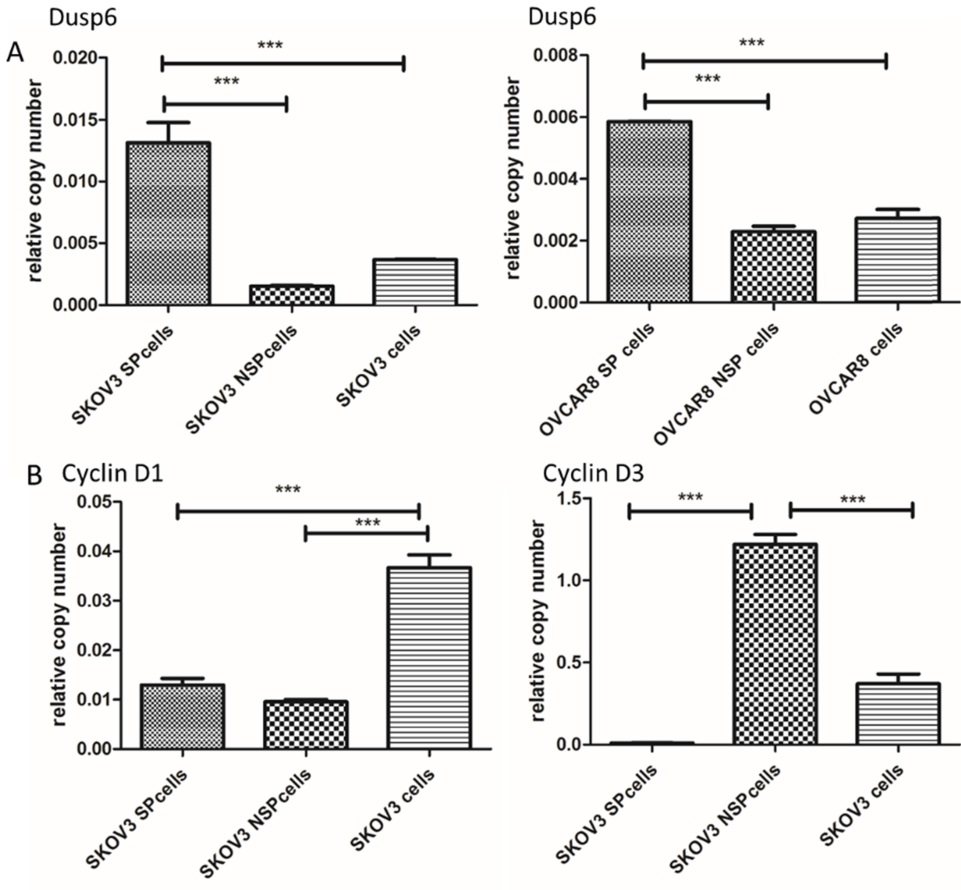
Differential expression of DUSP6 and G0/G1 cell cycle checkpoint regulating protein (CyclinD1, CyclinD3, CyclinE2) in SP and NSP cells. qRT-PCR was used to assess the expression level of DUSP6 (a), CyclinD1 (b,left) and CyclinD3 (b,right). Both SKOV3 and OVCAR8 cells, left and right panels in (a) respectively, were tested for DUSP6. Expression was assessed in SP, NSP, and original populations. *** indicates statistically significant difference (P < 0.001).

Among the differentially expressed genes, 5 (DUSP6, SOX9, DKK1, ARRDC3 and CSRNP2) were known to be associated with signaling pathways in cancer or cancer stem cells. DUSP6, SOX9, DKK1, ARRDC3 and CNRNP2 displayed gene expression fold changes of 6.15, 5.55, 4.46, 3.59 and 2.83, respectively. We chose DUSP6, which presented the highest expression change, for further study. To confirm that DUSP6 was overexpressed in SKOV3 and OVCAR8 ovarian cancer SP cells, we conducted a qRT-PCR analysis and the result showed that both SP cell populations expressed higher levels of DUSP6 mRNA expression (2 to 10-fold increase) compared with non-SP (NSP) cells (P<0.001) (Figure 3a). Thus, the RT-PCR analysis confirmed that expression of DUSP6 was higher in ovarian cancer SP cells, compared with NSP cells, a result consistent with the RNA‑seq analysis. SP cells have been reported to be mostly in G1 cell cycle arrest and stay quiescent 51. As cyclins (CyclinD1, CyclinD3 and CyclinE2) regulate the G0/G1 cell cycle checkpoint and play a pivotal role in G1/S phase transition^50^, we therefore examined expression of DUSP6 and these Cyclins in SKOV3 SP and NSP cells. Our result show that SKOV3 SP cells express lower levels on CyclinD3 mRNA compared with non-SP (NSP) cells (P<0.001). However, no differences in the expression of CyclinD1 or CyclinE2 were seen (Figure 3b).

### DUSP6 is overexpressed in chemotherapy-resistant ovarian cancer tissues

**Figure 4:**
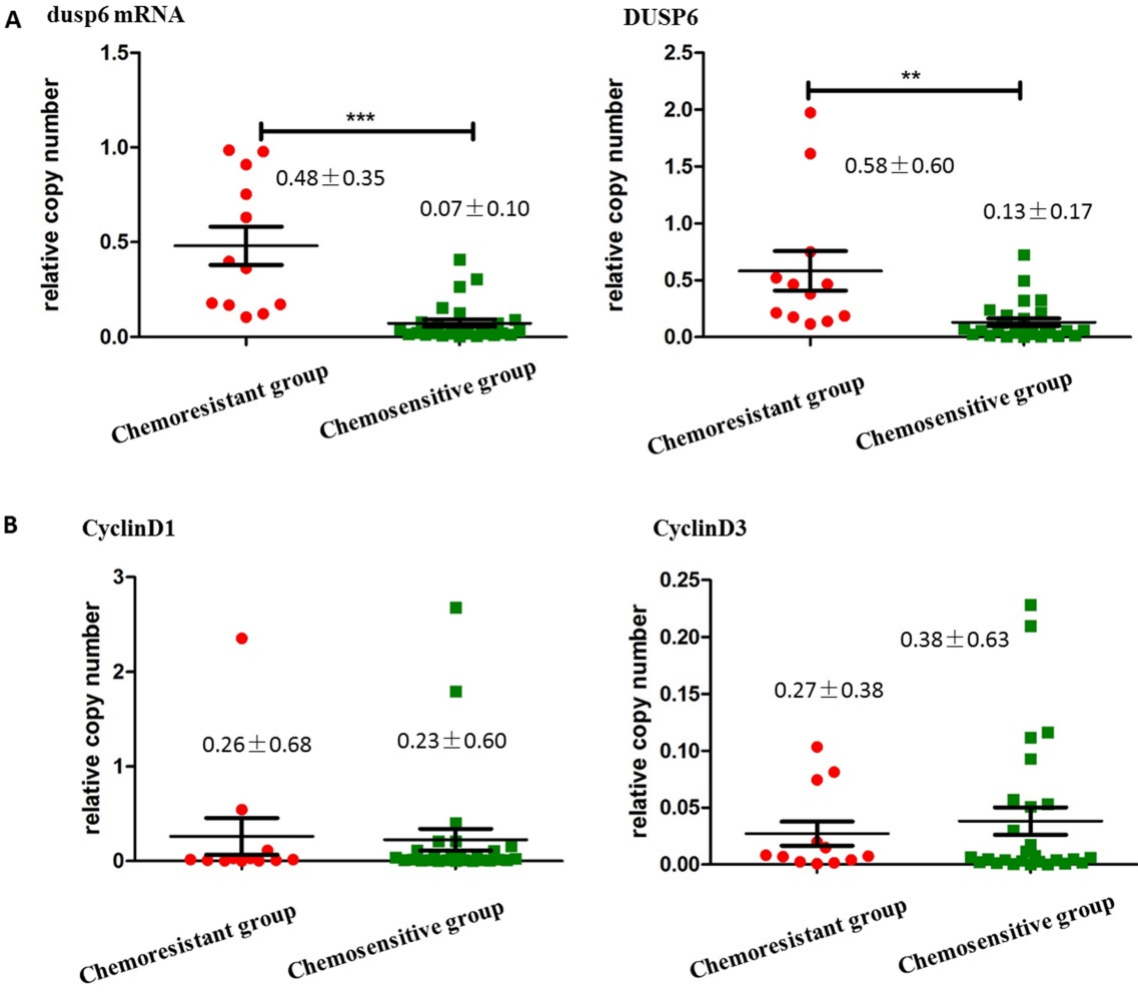
The expression of DUSP6, CyclinD1, CyclinD3 in ovarian cancer tissues. (a) qRT-PCR (left) and Western Blot (right) analysis of DUSP6 expression in chemotherapy-resistant (12 cases) and chemotherapy-sensitive (27 cases) tumors. (b) Western Blot analysis of CylinD1 (left) and CyclinD3 (right) expression in chemotherapy-resistant (12 cases) and chemotherapy-sensitive groups (27 cases). *** indicates statistically significant difference (P< 0.001).

To compare the expression levels of DUSP6 in ovarian cancer tissues with differing chemotherapy- sensitivity, tissue samples from 40 surgically resected stage III/IV primary ovarian cancers were collected for this study. Among these samples, one was from a patient that was complicated with renal carcinoma, and thus was excluded, leaving 39 enrolled tissue samples. Tissue samples were separated into two groups, chemotherapy-sensitive and chemotherapy- resistant, based on the clinical recurrence of the tumors. qRT-PCR and Western Blot analysis showed that the expression of DUSP6 in chemotherapy resistant tumors was higher than in chemotherapy- sensitive tumors (P<0.05). We also examined the expression of Cyclins D1, E2 and D3 in these two groups of tumors. Although expression of CyclinD3 was lower in the chemotherapy-resistant group, this difference was not statistically significant, and no differences in the expression of CyclinD1 or CyclinE2 were seen (Figure 4).

### DUSP6 is overexpressed in platinum-resistant ovarian cancer cells

**Figure 5:**
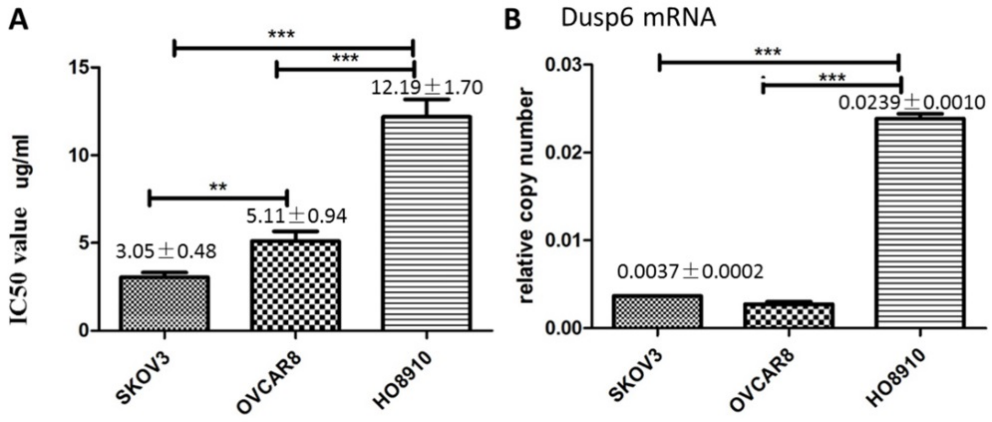
DUSP6 increases cell viability when treated with cisplatin. (a) MTS was used to measure cell viability and determine the IC50 value of cisplatin in SKOV3, OVCAR8 and HO8910 cell lines. (b) Expression level of DUSP6 mRNA. *** and ** indicate statistically significant differences, P<0.001 and P<0.05, respectively.

As cisplatin is commonly used in chemotherapy of ovarian cancer^3^, we explored the relationship between cisplatin resistance and DUSP6 expression levels in ovarian cancer cell lines. To assess cisplatin resistance, three ovarian cancer cell lines, SKOV3, HO8910 and OVCAR3, were treated with Cisplatin at different concentrations. Cell viability of the cell lines was measured by the MTS assay 48 hours after treatment, and the value of the half maximal inhibitory concentration (IC50) for each cell line was calculated using SPSS 19.0. The IC50 for cisplatin was higher for HO8910 cells than for the two other cell lines, suggesting that it is more resistant to platinum. Of the three cells examined, HO8910 also expresses the highest levels of DUSP6 mRNA (2- to 5-fold increase) (Figure 5), which suggests that DUSP6 contributes to platinum resistance.

### DUSP6 increases cisplatin resistance in ovarian cancer cells

**Figure 6:**
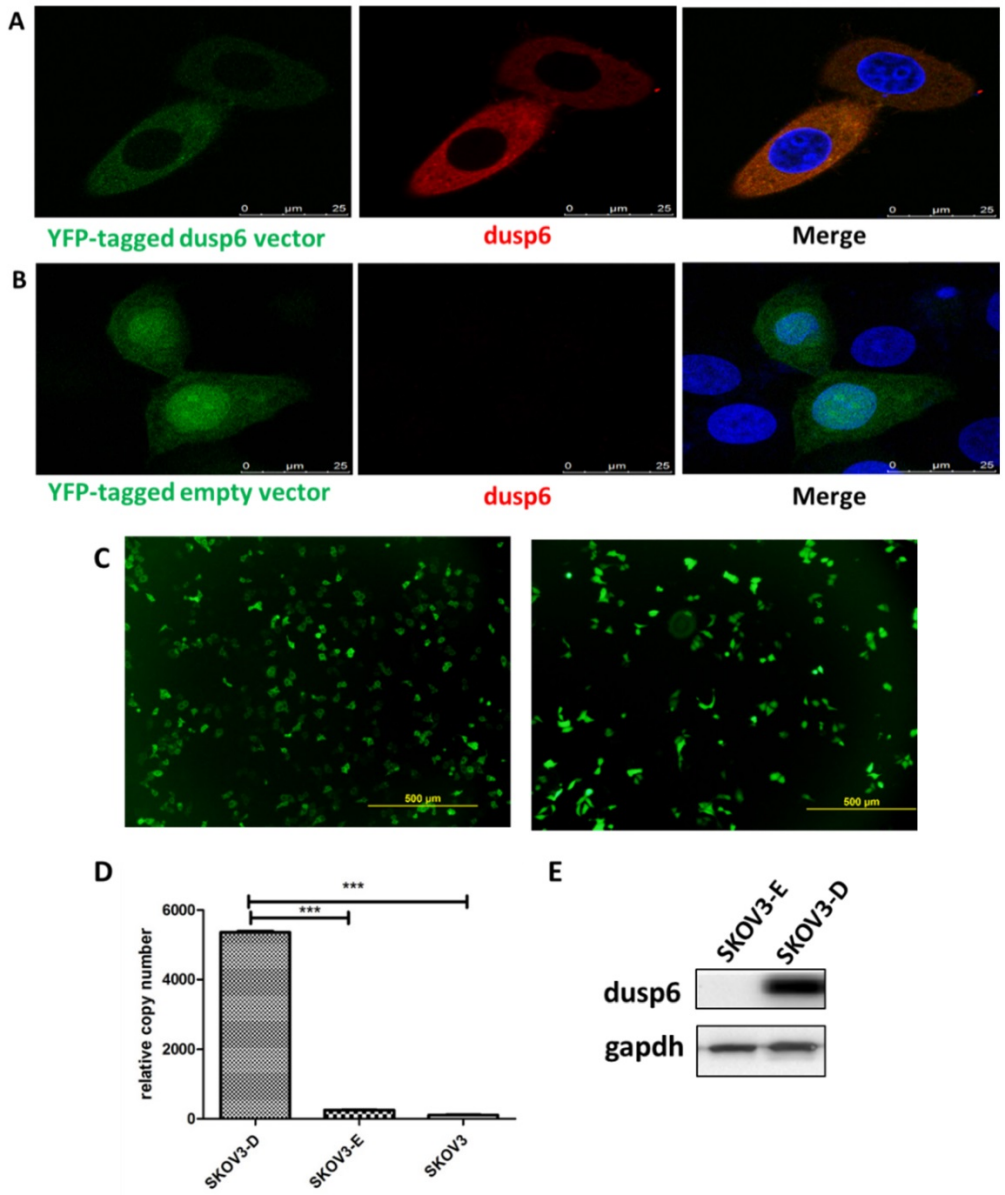
Overexpression of DUSP6 in SKOV3 cells. SKOV3 cells were transfected with YFP-tagged DUSP6-expressing construct and control cells were transfected with YFP-tagged empty vector. (a, b) Left panel shows confocal immunofluorescence to detect the expression of the linked yellow fluorescent protein (green) in YFP-tagged DUSP6-expressing construct (a) and YFP-tagged empty vector (b) transfected SKOV3 cells. Middle panel is detection of DUSP6 with 590 (red) fluorescent labeled DUSP6-specific antibody. Right panel is the merge of the two panels. Nuclei were stained with DAPI (blue). (c) Cells were viewed in 5 different fields under the microscope, with the total number of cells and the numbers of cells with yellow fluorescence counted. The transfection rate was calculated by the following formula, 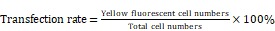 The transfection efficiencies were all above 75% in the SKOV3-D (left) and SKOV3-E (right) cells. (d) qRT-PCR and (e) Western Blot analysis DUSP6 expression in overexpressing (SKOV3-D), YFP empty vector (SKOV3-E), and parental (SKOV3) cells.

**Figure 7:**
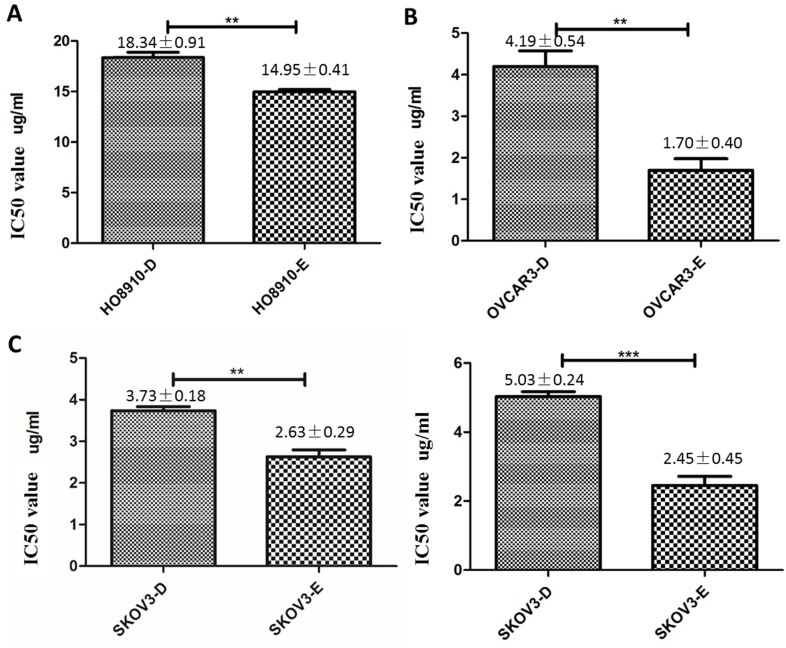
Overexpression of DUSP6 increases cisplatin resistance. HO8910 (a), OVCAR3 (b) and SKOV3 (c) cells were transfected with YFP-tagged DUSP overexpressing construct (-D) or YFP empty vector (-E) constructs. Transfected cells were treated with cisplatin and IC50 values were calculated. *** and ** indicate statistically significant differences, P<0.001 and P<0.05, respectively.

To further examine the role of DUSP6 in the chemotherapy-resistance to cisplatin we over- expressed DUSP6 in SKOV3 cells. A YFP-tagged DUSP6-expressing construct was used for overexpression, with expression examined via confocal immunofluorescence, qRT-PCR and Western Blot analysis. The transfection efficiencies were all above 75% in the SKOV3-D and SKOV3-E cells (Figure 6c). Compared to the YFP-tagged empty vector, SKOV3 cells transfected with the DUSP6-YFP-tagged construct showed expression, via immunofluorescence (Figure 6a, b), qRT-PCR (Figure 6d) and Western blot (Figure 6e), of DUSP6 that was increased above the endogenous level. We then examined the cisplatin resistance of the transfected cells. The IC50 values of DUSP6-overexpressing ovarian cancer cells were significantly elevated above control cells transfected with YFP-tagged empty vector (P<0.05). Moreover, after sorting cells via their YFP tag, purified DUSP6-overexpressing SKOV3 cells were also found to have a higher IC50 value compared to control cells that were purified in the same way (P<0.001) (Figure 7).

### DUSP6 is a negative regulator to ERK1/2 in ovarian cancer cells

**Figure 8:**
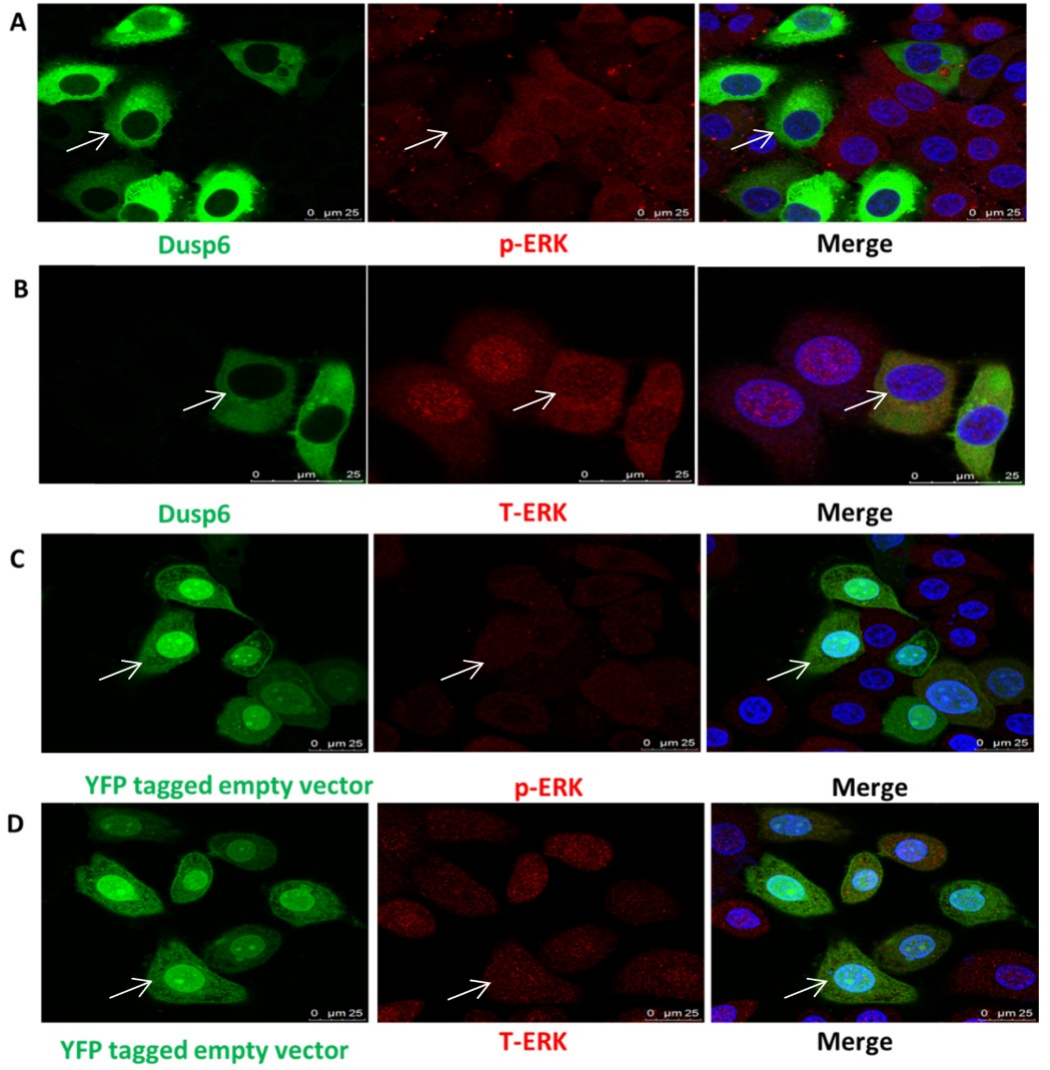
Overexpression of DUSP6 reduces pERK translocation to the nucleus. SKOV3 cells transiently transfected with pcDNA3.1-DUSP6-YFP plasmid (a, b) and pcDNA3.1-YFP plasmid (c, d) respectively, using immunofluorescence to visualize the change of subcellular localization of p-ERK and T-ERK.YFP were visualized using secondary Alexa 488 (green) or 590 (red) fluorescent labeled antibody and DAPI (blue). The same cell in the green (DUSP6 or empty vector), red (p-ERK) and merge panels is identified by an arrow.

**Figure 9:**
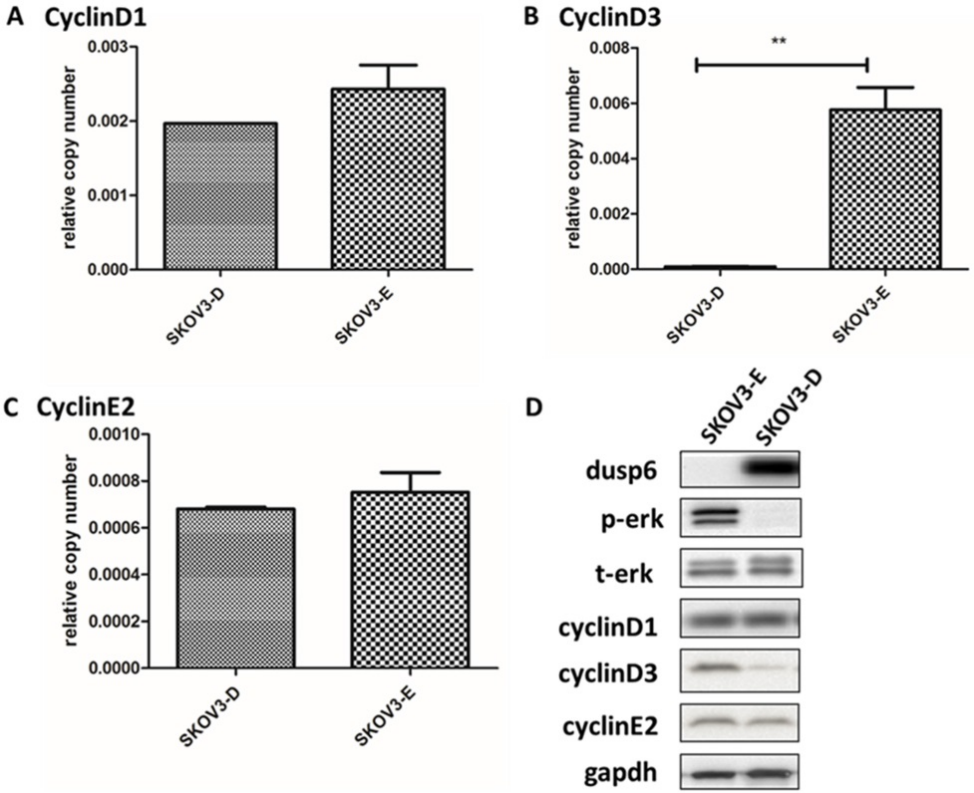
Overexpression of DUSP6 decreases CyclinD3 expression. SKOV3 cells were transiently transfected with pcDNA3.1-DUSP6-YFP plasmid (SKOV3-D) or empty pcDNA3.1-YFP vector (SKOV3-E). Expression of (a) CyclinD1, (b) CyclinD3 and (c) CyclinE2 was assessed by qRT-PCR and (d) by Western blot. ** indicates statistically significant difference, P<0.05.

As DUSP6 regulates ERK1/2 function in cells^36^-^49^ we examined the effect of the overexpression of DUSP6 in SKOV3 cells. Analysis of the abundance of ERK1/2 by immunofluorescence demonstrated that overexpression of DUSP6 in SKOV3 cells remarkably reduced the expression levels of phospho- ERK1/2 and may prevent cytoplasmic p-ERK from translocating to the nucleus (Figure 8). Analysis of cyclin mRNA levels by qRT-PCR analysis showed that DUSP6-overexpressing SKOV3 (SKOV3-D) cells express lower levels of cyclinD3 mRNA (2- to 10-fold change) compared with control (SKOV3-E) cells (p<0.005). However, no difference in the expression level of cyclinD1 or cyclinE2 mRNAs were seen between the DUSP6 overexpressing and control SKOV3 cells. These results suggest that DUSP6- overexpressing SKOV3 cells reduced the expression levels of phospho-ERK1/2, which then leads to decreased transcriptional activity of its downstream effector cyclinD3 (Figure 9).

### Overexpression of DUSP6 may lead to G0/G1 cell cycle arrest

**Figure 10:**
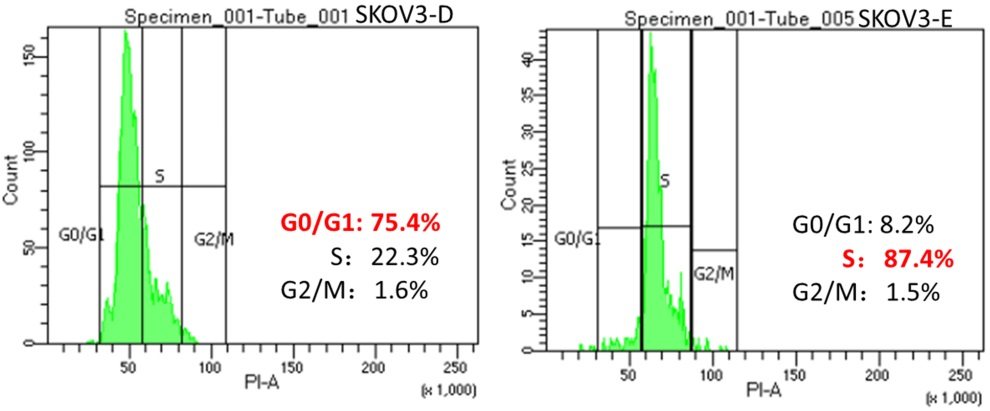
Changes in the cell cycle with overexpression of DUSP6. SKOV3 cells were transiently transfected with pcDNA3.1-DUSP6-YFP plasmid (SKOV3-D, left panel) or empty pcDNA3.1-YFP vector (SKOV3-E, right panel). Proportion of cells in the different phases of the cell cycle was assessed by FACS analysis.

To confirm the hypothesis that DUSP6 increases the proportion of cells in G0/G1 phase of the cell cycle in ovarian cancer cells by negatively regulating the ERK signaling pathway, which leads to cellular quiescence and therefore causing chemotherapy- resistance, we use FACS to conduct a cell cycle analysis in DUSP6 overexpressing and control SKOV3 cells. Our results show that the DUSP6-overexpressing SKOV3 (SKOV3-D) cells have a predominance of cells in G0/G1 phase, whereas control (SKOV3-E) cells are mostly in the S phase (Figure 10). This shows that overexpression of DUSP6 directly regulates the cell cycle.

## Discussion

Typically, ovarian cancer patients initially respond well to surgical cytoreduction and chemotherapy, with chemotherapy alone often yielding several logs of tumor cytoreduction, but seldom is it a cure^1^. The majority of ovarian cancer patients with advanced disease eventually redevelop tumors that are chemotherapy resistant^2^-^3^. It has been hypothesized that the failure to completely eradicate ovarian cancers is attributed to the existence of cancer stem-like cells that lead to recurrence and resistance to chemotherapy^4^. Cancer stem-like cells, like somatic stem cells, are thought to have the properties of relative quiescence, ability for self-renewal, the capacity to induce tumorigenesis, and resistance to chemotherapeutic agents^4^-^5^. Recent studies have indicated that SP cells isolated from human ovarian cancer cells, using Hoechst dye exclusion after flow cytometry, have stem cell-like characteristics^11^,^13^. These cells provided us with a new entrance to explore the mechanisms leading to chemotherapy- resistance.

In our study we had identified an association between the DUSP6 gene and chemotherapy- resistance in ovarian cancer SP cells, by using qRT‑PCR, we confirmed that DUSP6 expression was higher in ovarian cancer SP cells, and in other chemotherapy-resistant ovarian cancer cell lines and tissue samples, than in non-chemotherapy resistant cells (Figure 3a, 4a). Additionally, using the MTS assay we demonstrated that the overexpression of DUSP6 lead to an increase in the IC50 to cisplatin in ovarian cancer cells (Figure 5). Our results indicate that the expression of DUSP6 has a close association with drug resistance. High expression of DUSP6 may act as a positive indicator for chemotherapy-resistance and thus suggests it might have predictive value for EOC chemotherapy-resistance. We attempted to replicate these findings using data from the TCGA database. Among 396 ovarian cancer tissue samples reported in this database, no difference (P=0.766) in the expression of DUSP6 was found between chemotherapy-resistant (42 samples) and chemotherapy- sensitive (284 samples) patients. However, this result is of limited value due to the difficulty in defining the chemotherapy-sensitive group. Chemotherapy-sensitivity was not diagnosed in the patients in the TCGA database; thus, we could only use progression-free time to separate these two groups. These differences in defining the chemotherapy-sensitive and resistant groups between our patients and the TCGA database may explain the different observations concerning the levels of DUSP6 expression.

**Figure 11:**
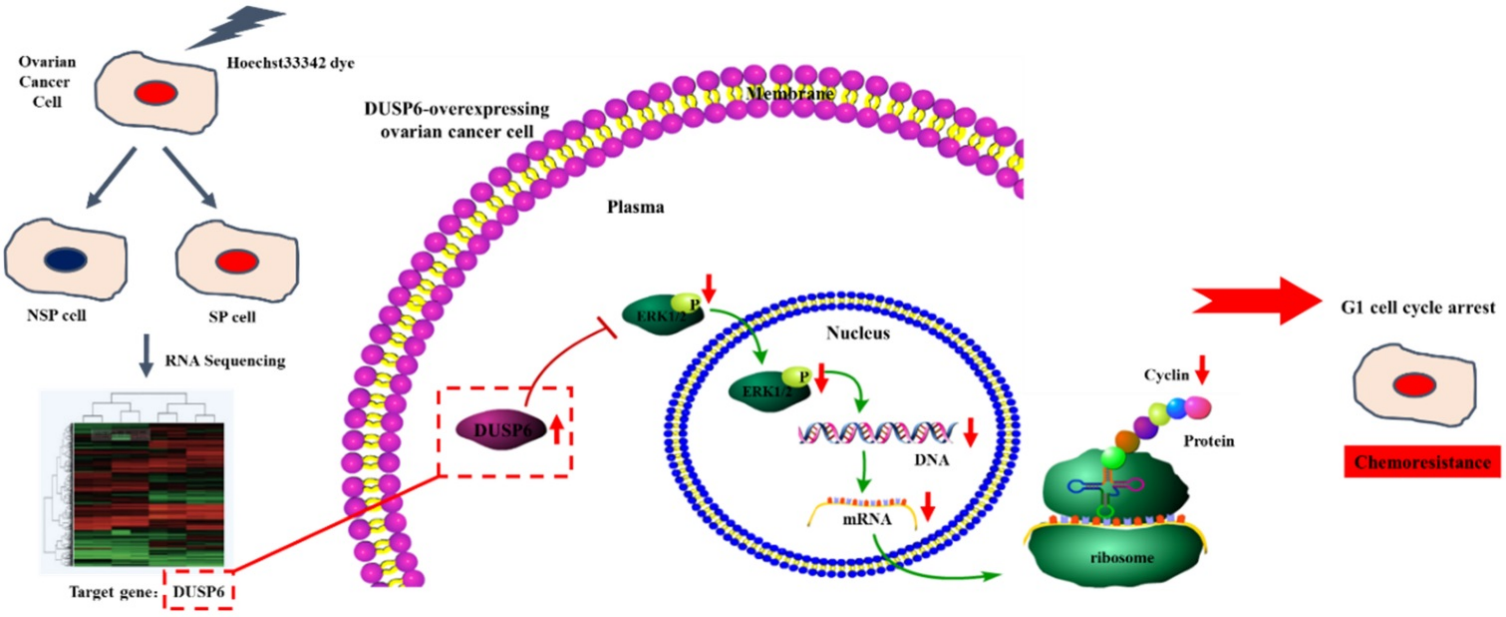
Possible mechanism for the enhancement of chemotherapy-resistance by DUSP6-overexpressing ovarian cancer cells. Based on RNA-sequencing, with verification by qRT-PCR, DUSP6 is overexpressed in ovarian cancer SP cells. Our study demonstrated that DUSP6-overexpressing SKOV3 cells possess lower levels of CyclinD3. As DUSP6 is a known negative regulator of ERK1/2, we revealed that overexpression of DUSP6 decreases phospho-ERK1/2 levels in parallel with attenuated cyclin D3 expression. CyclinD3 plays an important role in G1/S phase transition and decreased levels are linked with G0/G1 cell cycle arrest. Since SP cells are predominantly in G1 arrest, and chemotherapy-resistant, we demonstrated that DUSP6 increases the G0/G1 phase ratio in ovarian cancer cells by negatively regulating ERK signaling, which links cellular quiescence to chemotherapy-resistance.

Our study also suggests that the overexpression of DUSP6 in ovarian cancer SP cells is accompanied with decreased expression level of CyclinD3 (Figure 3b). Similar findings have also been recently reported by other groups^52^. DUSP6-overexpressing SKOV3 cells also express lower levels of CyclinD3 (Figure 9). As DUSP6 is known to be a negative regulator of ERK1/2^32^-^37^, we examined the levels of phosphor- ERK1/2 in these cells. Our study revealed that overexpression of DUSP6 decreased phospho- ERK1/2 levels in parallel with attenuated cyclin D3 expression (Figure 9b). CyclinD3 plays an important role in G1/S phase transition and decreased levels are linked with G0/G1 cell cycle arrest^25^,^50^. Since SP cells are predominantly G1 cell cycle phase arrested^51^, and chemotherapy-resistant, we hypothesized that DUSP6 may increase the G0/G1 phase ratio in ovarian cancer cells by negatively regulating the ERK signaling pathway, which then may lead to cellular quiescence and therefore chemotherapy-resistance. To test this hypothesis, we use FACS to conduct a cell cycle analysis, which showed that DUSP6-overexpressing SKOV3 cells were predominantly G1 cell cycle phase arrested. These results indicate that the overexpression of DUSP6 enhances the chemotherapy-resistance property of ovarian cancer by promoting G1 cell cycle arrest. Therefore, DUSP6 may play a role in EOC chemotherapy-resistance and is potentially a chemotherapy-sensitizing target (Figure 11).

Consistent with our findings, studies of different types of solid tumors, including ovarian, breast, pancreatic, hepatocellular, esophageal, prostate, and lung carcinoma, reported that DUSP6 is under- expressed in highly proliferating tumor cells compared with normal cells^28^,^29^,^32^-^50^. This was thought to be due to DUSP6 acting as an ERK1/2- specific negative regulator and suppression of the transcriptional activity of its downstream factors. However, expression of DUSP6 increases as tumor proliferating activity decreases^32^-^34^. In lung cancer^35^-^36^ and glioblastoma^44^-^45^, research has revealed that the proportion of cell in S phase is decreased and those in G1 phase increased in DUSP6- overexpressing tumor cells. This suggests that DUSP6 functions as a growth suppressor by preventing cell cycle progression and thus keep cellular quiescence. Cisplatin is one of the most potent antitumor agents used in cancer^6^-^7^, though its antitumor effect cell cycle phase nonspecific^8^, however, highly proliferating (S phase predominant) cells are more sensitive than those that are quiescent (G0/G1phase predominant)^8^,^51^. Some G1 phase predominant DUSP6- overexpressed tumor cells are considered to be cisplatin-resistant^44^,^45^. In accordance with our results, studies in glioblastoma also showed that DUSP6 overexpression increased resistance to cisplatin by regulating the ERK signaling pathway^34^-^45^.

While there have been only few studies concerning DUSP6 and ERK signaling pathway in ovarian cancer, one study presented a result that is contradictory to ours, as it showed that the loss of DUSP6 enhances chemotherapy-resistance in ovarian cancer cells^37^. Their study used a short hairpin RNA to knock-down the expression of the endogenous DUSP6 gene in A2780s ovarian cancer cells, which were then treated with two concentration of cisplatin (5 and 10 uM). While their study only used one cell line, here we were able to replicate our results in three different cell lines.

To verify the crucial role of DUSP6 in this cellular regulation process, we should also study the effects of under-expressing or silencing DUSP6. We have initiated studies to examine the expression of p-ERK, and its downstream factors CyclinD1, D3 and E2, when DUSP6 is under-expressed, and its effect on chemotherapy-sensitivity. In addition, a major limitation of our study is identification of the mechanism linking DUSP6 induction to G1 cell cycle phase arrest. Whether only the ERK signaling pathway regulates this process, or whether other cellular signaling pathways have roles requires further study. To address this, we could block ERK signaling using specific inhibitors to determine whether this inhibits DUSP6 regulation of the cell cycle. If blockage of ERK signaling does not lead to a loss of DUSP6 mediated regulation of cell cycle and chemotherapy-sensitivity, this would suggest that other pathways are involved, such as the Wnt and Akt pathways. Studies concerning the roles of these pathways in connection with DUSP6 and regulation of cell-cycle are required.

In conclusion, our findings revealed the enhancement of chemotherapy-resistance and the predominance of G1 cell cycle phase arrest in DUSP6-overexpressing ovarian cancer cells. This suggests that overexpression of DUSP6 may promote chemotherapy-resistance through the negative regulation of the ERK signaling pathway, which increases the G0/G1 phase ratio among ovarian cancer cells leading to their cellular quiescence. Taken together, these results suggest that DUSP6 should be considered as a potential target for predicting and regulating the platinum chemotherapy-resistance properties of advanced ovarian epithelial cancers. Further study is needed to better understand the molecular mechanism regulated by DUSP6, which may assist the development of new therapeutic interventions for chemotherapy-resistance.

## Supplementary Material

Supplementary tables.
