# Supplementary material for: Overexpression of DUSP6 enhances chemotherapy-resistance of ovarian epithelial cancer by regulating the ERK signaling pathway
Source: J Cancer. 2020 Mar 4;11(11):3151–64. doi: 10.7150/jca.37267 (PMC7097933; doi:10.7150/jca.37267)
Supplement: Supplementary file 1 — Supplementary tables. [file jcav11p3151s1.pdf]

Supplementary Table 1

Table S1 Clinicopathological characteristics of the patients in this study

**Characteristic**	**Chemotherapy resistant group†**	**Chemotherapy sensitive group†**
Sample counts	12	27
Age(median(range))	52 (40-76)	58 (34-72)
Tumor size>5cm (yes/no)	10/2	19/8
Massive Ascites >500ml (yes/no)	6/6	7/20
Liver metastasis (yes/no)	3/9	1/27
Preoperative CA-125 level (mIU/ml, median (range))	1016 (158-5856)	539 (94-6455)
FIGO stage (IIIC/IV)	5/7	24/3
Residual disease (R0/R1/R2)‡	1/5/6	6/15/6

Notes: †Chemotherapy resistance was defined as recurrence within 6 months of achieving complete recovery after the initial treatment or disease progression during chemotherapy. Chemotherapy sensitive patients were defined as having recurrence more than 6 months after achieving complete recovery after the initial treatment.

‡R0 refers to no residual disease; R1 refers to residual disease with tumors ＜ 1cm; R1 refers to residual disease with tumors ≥ 1cm

Supplementary Table 2

Table S2 Primers for reverse transcription-quantitative polymerase chain reaction.

Gene Name	Primer sequence (5'-3')	
	Forward	reverse
actin	GAAATCGTGCGTGACATTA	AGGCAGCTCGTAGCTCTT
DUSP6	GAGTCTGACCTTGACCGAGACCCC	TTCCTCCAACACGTCCAAGTTGGT
cyclinD1	GTCCTACTTCAAATGTGTGCAGAAG	GGGGATGGTCTCCTTCATCTTAGAG
cyclinD3	GACCATCGAAAAACTGTGCATCTAC	CATGGCAAAGGTATAATCTGTAGCA
cyclinE2	AACTACCCAGGATGTCAAAAAAAGA	ATCAGGCAAAGGTGAAGGATTAATA
